# Periodontal Treatment of Norwegian Patients With Rare Diseases: A Commentary

**DOI:** 10.1016/j.identj.2023.07.009

**Published:** 2023-09-23

**Authors:** Øystein Fardal, Irene Skau, Jostein Grytten

**Affiliations:** a Private practice, Egersund, Norway; b Institute of Community Dentistry, University of Oslo, Oslo, Norway; c Institute of Education for Medical and Dental Sciences, University of Aberdeen, Aberdeen, Scotland; d Division of Obstetrics and Gynaecology, Akershus University Hospital, Lørenskog, Norway

## Introduction

Periodontal treatment followed by maintenance therapy has been shown to be effective in reducing tooth loss for patients with periodontal diseases.^1^ However, a minority of patients lose many teeth in spite of treatment. Many of these patients have added risk factors such as systemic diseases, genetic predispositions, and/or smoking.^2^ A number of rare diseases (RDs) can have detrimental effects on the periodontal tissues and cause tooth loss.

In the US, a disease is classified as “rare” when fewer than 1 in 1500 people is affected, whilst in Europe it is 1 in 2000.^3^^,^^4^ In the US, 30 million patients are living with RDs, and worldwide, approximately 350 million are living with RDs. Most of the RDs are thought to be innate, with a strong genetic base. Due to the rareness, clinical research is difficult and the mechanisms and aetiologies remain unknown for many of the RDs. In addition, it is difficult for pharmaceutical companies to find a commercial basis for developing treatments for only a few patients, so presently less than 5% of the approximately 8000 RDs have effective medical treatments.^5^ A number of patients with RDs receiving little or no treatment thus have the extra burden of being susceptible to periodontal diseases.

Most oral clinicians will at some stage encounter patients with rare diseases. It is important not only to be aware of which RDs are associated with periodontal complications but also to know which ones are likely to need extensive periodontal treatments. It is important to have knowledge about the disease itself and the mechanisms involved to avoid complications during treatment. In addition, the clinicians can help diagnosing RDs based on recognisable clinical features. Early detection can be vital for patients’ survival.^6^

### Increased susceptibility to periodontal diseases

The mechanisms behind the periodontal complications in RDs are mainly related to immunologic disorders, disorders of bone metabolism, neoplastic diseases, and diseases that affect the oral mucosa and gingival tissues.^7^^,^^8^ The most common immunologic disorders are Papillon-Lefèvre syndrome, Ehlers-Danlos syndrome, plasminogen deficiency, Cohen syndrome, and neutropenia. Diseases involving bone metabolism include hypophosphatasia, hyperparathyroidism, and hypophosphatemic rickets. Neoplastic diseases include neurofibromatosis, Langerhans cell histiocytosis, and Gorham-Stout disease. Diseases of the oral mucosa are epidermolysis bullosa and Kindler syndrome.

A study from Germany reported that 14% of the 541 RDs with orofacial manifestations also had periodontal complications.^9^ These included gingivitis, periodontitis, gingival hyperplasia, and haemorrhagic diatheses.

Little attention has been given to the fact that physical and mental disorders which are part of some RDs can affect oral hygiene performance and, in this way, make the patients more susceptible to periodontal diseases.

Most of the information on the periodontal problems in RDs is obtained from case reports and narrative reviews.^7^^,^^8^ Since these case reports and reviews report nearly exclusively on cases which have periodontal complications, it gives the impression that most or all patients with certain RDs should expect complications. It is not known how many of the RDs need periodontal treatment, nor is it known whether all patients within a certain RD group need treatment.

Rare diseases present a wide range of alterations and symptoms that vary not only from one disease to another but also from patient to patient according to the degree of affectation and evolution. Some of the patients have only minor signs and symptoms.^10^ Periodontal diseases and certain systemic disorders share similar genetic and/or environmental aetiologic factors, and affected individuals may show manifestations of both diseases. It seems logical that periodontal manifestations and alterations should express a similar diversity pattern where not all patients are necessarily affected within an RD group. There is evidence of such partial expression of the periodontal complications in some RDs, for example, in Papillon-Lefèvre syndrome.^11^

### Periodontal treatments for RDs

Due to the low number of patients and a lack of reporting on treatments in many of the case reports, it has been impossible to establish the prevalence and extent of periodontal treatments performed for patients with RDs.

Meaningful analyses on the treatment require big data, preferably on a national scale. So far, there has been a scarcity of relevant big data which explains the paucity of research dealing with the periodontal treatments of RDs.

The Norwegian National Insurance Scheme has, for over 5 decades, provided a major contribution towards periodontal therapy for all citizens.^12^ The scheme pays for most of the treatment for RDs patients, and the scheme has data on all these patients. The National Insurance Scheme has identified 357 RDs which qualify for added financial support for medical and dental care.^13^ An analysis of the complete dataset from the Norwegian National Insurance Scheme would provide a unique opportunity to investigate the levels of periodontal treatments performed for the various RDs.

### An observational study using population register data from Norway

The Norwegian Directorate of Health made the database from the National Health Economics Administration for 2017-2020 available to the Department of Community Dentistry, University of Oslo. The main data for this study were extracted from 2019, using data from 2017 and 2018 to verify the representativeness. The research was approved by The Norwegian Regional Committee for Medical and Health Research Ethics (reference number 28420).

Prevalence of the total periodontal treatment for all RDs patients and prevalence for each of the RDs groups were assessed.

A total of 8467 patients from 253 RDs (64.1% females and 35.9% males; average age, 52.6 years; range 18–94 years; SD, 16.2 years) were identified, and 867 (10.2%) of these (66.9% females and 33,1% males; average age, 57.1 years; range, 19–89 years; SD, 13.7 years) from 135 RDs were treated for periodontal diseases. There was a wide range in the number of patients identified in each of the RDs (1–709).

The results showed that an average of 10.2% of the patients with RDs were treated for periodontal diseases. Table 1 shows all the RDs with percentages of patients receiving treatment. The medical complications that rated highest in terms of periodontal treatments were as follows: premature fusion of sutures, bone growth and development disorders, neutropaenia, skin and connective tissue disorders, osseous dysplasia, chromosomal defects, inflammatory diseases including blood vessel inflammation, digestive system disorders, autoimmune adrenocortical insufficiencies, increased numbers of mast cells, hypoparathyroidism, and blood disorders.

**Table 1:** Number of patients with rare diseases, number of patients treated for periodontal diseases, and percentage patients treated for periodontal diseases.

Rare disease	No. of patients with rare diseases	No. of patients with rare diseases treated for periodontal diseases	Treated for periodontal diseases (%)
Achalasia	69	9	13.0
Achondroplasia	25	2	8.0
Acromegaly	215	13	6.0
Acute intermittent porphyria	104	7	6.7
Acute porphyria	32	6	18.8
Adrenogenital syndrome	43	2	4.7
Alport syndrome	2	1	50.0
Amyloidosis, primary and secondary	38	5	13.2
Amyotrophic lateral sclerosis	47	7	14.9
APS type 1 and type 2	172	22	12.8
Arthrogryposis multiplex congenita	46	8	17.4
Behçet's syndrome	27	1	3.7
Benign hereditary chorea	3	1	33.3
Bethlem myopathy	5	1	20.0
Blepharophimosis-ptose-epicanthus inversus (BPES)	2	1	50.0
Branchiootorenal syndrome	5	1	20.0
Bullous mucous membrane pemphigoid	42	7	16.7
Cerebellar ataxia	65	10	15.4
Cherubism	12	1	8.3
Charcot-Marie-Tooth disease	444	40	9.0
Chronic mucocutaneous candidiasis	5	1	20.0
Chronic neutropenia	13	1	7.7
Churg-Strauss syndrome	54	9	16.7
Cleidocranial dysplasia	15	1	6.7
Complicated hereditary spastic paraparesis	52	4	7.7
Congenital erythropoietic porphyria	3	1	33.3
Congenital muscular dystrophy	6	1	16.7
Cowden disease (multiple hamartoma syndrome)	43	4	9.3
Crouzon syndrome	14	4	28.6
Currarino syndrome	6	1	16.7
Cystic fibrosis	112	9	8.0
Darier disease	23	5	21.7
Dercum's disease (adiposis dolorosa)	3	1	33.3
Dermatomyositis	76	6	7.9
Duchenne muscular dystrophy	5	1	20.0
Dyskeratosis congenita	1	1	100.0
Ectodermal dysplasia	68	7	10.3
Ehlers-Danlos syndrome	709	70	9.9
Epidermolysis bullosa dystrophic junctional	34	3	8.8
Epiphyseal dysplasia	11	1	9.1
Erdheim-Chester disease	10	4	40.0
Erythropoietic porphyria	4	1	25.0
Fabry disease	52	5	9.6
Facioscapulohumeral muscular dystrophy	17	3	17.6
Familial adenomatous polyposis	8	1	12.5
Fibrous cemento-osseous dysplasia	24	5	20.8
Freeman Sheldon syndrome	5	2	40.0
Friedreich's ataxia	18	3	16.7
Glycogenesis	6	2	33.3
Gorlin syndrome	45	4	8.9
Granulocytopenia	7	2	28.6
Hallervorden-Spatz syndrome (PKAN)	20	2	10.0
Hemihypertrophy	3	1	33.3
Haemophilia A and B	191	18	9.4
Hereditary angioedema	72	6	8.3
Hereditary congenital facial paresis	9	2	22.2
Hereditary immune failure	79	8	10.1
Hereditary neuropathy	60	2	3.3
Huntington's disease	110	11	10.0
Hurler syndrome	2	1	50.0
Hypogammaglobulinemia	149	15	10.1
Hypoparathyroidism	98	12	12.2
Hypophosphatemic rickets	26	3	11.5
Hypopituitarism	117	14	12.0
Ichtyosis	8	1	12.5
IgA and IgG deficiency (both)	68	5	7.4
Inclusion body myositis	28	5	17.9
Incontinentia pigmenti	18	2	11.1
Intestinal lymphangiectasia	3	2	66.7
Klippel-Feil syndrome	6	2	33.3
Klippel-Trénaunay-Weber syndrome	14	2	14.3
Langerhans cell histiocytosis	19	1	5.3
Laurence-Moon-Bardet-Biedl syndrome	19	2	10.5
Leri-Weill dyschondrosteosis	52	6	11.5
Leukodystrophy	10	1	10.0
Limb-girdle syndrome	130	10	7.7
Lipodystrophy	4	1	25.0
Loeys-Dietz syndrome	24	2	8.3
Lymphangioma	9	2	22.2
Maple syrup urine disease	3	1	33.3
Marfan syndrome	167	17	10.2
Mastocytosis	80	10	12.5
MELAS syndrome	31	4	12.9
Melnick-Needles syndrome	3	1	33.3
MERRF syndrome (myoclonus epilepsy with ragged red fibers)	4	3	75.0
Mixed systemic connective tissue disease	144	13	9.0
Mucopolysaccharidosis	5	1	20.0
Multiple endocrine neoplasia type 1 (MEN-1)	19	3	15.8
Myasthenia gravis	285	33	11.6
Myotonic dystrophy	209	31	14.8
Necrotising vasculitis	31	1	3.2
Neurofibromatosis 2	86	10	11.6
Neutropaenia	29	7	24.1
Noonan syndrome	31	3	9.7
Oculo-auriculo-vertebral spectrum	13	2	15.4
Oesophageal atresia	32	1	3.1
Orofacialdigital syndrome (OFD)	9	1	11.1
Orofacial granulomatosis	309	25	8.1
Osler-Weber-Rendu syndrome	268	19	7.1
Osteogenesis imperfecta	153	11	7.2
Pachydermoperiostosis	1	1	100.0
Papillon-Lefèvre syndrome	3	1	33.3
Paramyotonia congenita and hyperkalemic periodic paralysis	2	1	50.0
Pemphigus all types	81	9	11.1
Pierre Robin sequence	12	2	16.7
Poland syndrome	5	2	40.0
Porphyria variegata	18	2	11.1
Primary lateral sclerosis	10	2	20.0
Pseudohypoparathyroidism	11	1	9.1
Pseudopseudohypoparathyroidism	3	1	33.3
Pyoderma gangrenosum	14	2	14.3
Rare chromosome or genetic defects	58	10	17.2
SAPHO syndrome	32	8	25.0
Scapulo-humoral musculardystrophy	26	3	11.5
Shwachman-Diamond syndrome	3	1	33.3
Scleroderma	555	73	13.2
Serious factor V deficiency	16	1	6.3
Serious factor VII deficiency	15	1	6.7
Serious function defects in thrombocytes	37	5	13.5
Seropositive juvenile rheumatoid arthritis	24	2	8.3
Sjögren-Larsson syndrome	297	24	8.1
Smith-Magenis syndrome	2	2	100.0
Spinal muscular atrophy	24	2	8.3
Spondyloepiphyseal/-metaphyseal dysplasia	11	2	18.2
Stickler syndrome	11	1	9.1
Sturge-Weber syndrome	5	1	20.0
Treacher Collins syndrome	27	2	7.4
Trichorhinophalangeal syndrome	15	1	6.7
Tuberous sclerosis	45	3	6.7
Tumoral calcinosis	3	2	66.7
Usher syndrome	48	6	12.5
Velocardiofacial syndrome	27	1	3.7
Von Hippel-Lindau syndrome	7	1	14.3
Wegener's granulomatosis	379	46	12.1
Williams syndrome	8	1	12.5

In addition to these disorders, relatively high levels of periodontal treatments were observed to be performed for patients with RDs with debilitating neuromuscular disorders such as facioscapulohumeral muscular dystrophy, Friedreich's ataxia, cerebellar ataxia, and myotonic dystrophy. These types of RDs are not usually included as predisposing diseases for periodontal complications; however, it is possible that they may result in a reduced ability to perform oral hygiene and thus make these patients more prone to periodontal diseases. In addition, nerve disorders may result in a reduced salivary flow.

Fibrous cemento-osseous dysplasia has so far been regarded as a peri-apical disease, but the present study shows that it also required periodontal treatment.

Even with national population register data, there were some RDs that were so rare that they were noted to affect only a few patients. It is difficult to perform meaningful assessments for these RDs, as they may not give a correct reflection of the proportions receiving treatments. For example, with only one patient in an RD group, the result will be either 100% or 0%. Only the RD groups with 10 or more patients were therefore included in Table 2, which shows the RDs with the highest levels of periodontal treatments with short descriptions.

**Table 2:** Rare diseases with the highest treatment levels (>10 patients in each rare disease group).

Rare disease	Percentage treatment	Comments
1. Crouzon syndrome	28.6	A premature fusion of certain skull bones (craniosynostosis) caused by a specific genetic mutation (fibroblast growth factor receptor 2). The mechanism(s) behind the periodontal complication has not been documented. It is plausible that crowding of teeth and a narrow palate may be contributory. In addition, it has been reported that dental pulp pluripotential cells from patients with Crouzon have a different mRNA and protein expression related to cellular differentiation than pulp cells from normal persons. This is characterised by a higher expression of osteocalcin and a lower phosphorylation rate.
2. SAPHO syndrome	25.0	A chronic inflammatory disorder of bone, joint, and skin characterised by synovitis, osteitis, hyperostosis, and enthesitis. It is characterised by pain, swelling, and tenderness in the affected areas. The pathogenesis of the SAPHO syndrome is not defined, but it is likely to include a combination of genetic, infectious, and immunologic components leading to the activation of the innate and cell-mediated immune systems. It is classified as an autoinflammatory disorder. The mechanism(s) behind the periodontal complications have not been documented; however, chronic osteomyelitis with diffuse sclerosing of the mandible has been described.
3. Neutropaenia	24.1	A deficiency in the immune response due to low neutrophil count. The following defect resulting in the poor immune response has been proposed: The neutrophils are deficient in the antibacterial peptide cathelicidin LL‐37 and have reduced concentrations of the human neutrophil peptides 1–3 (HNP1‐3; α‐defensins).
4. Darier disease (keratosis follicularis/dyskeratosis follicularis)	21.7	A skin condition characterised by wart-like blemishes on the body. Fifty percent are reported to have oral manifestations. The palate is the most affected area, followed by the gingiva, oral mucosa, and tongue. In addition, 30% of patients have intermittent sialadenitis of the parotid glands. The disease is caused by mutations in the *ATP2A2* gene that encodes the sarco/endoplasmic reticulum Ca2+-adenosine triphosphate (ATP)ase pump (SERCA2). This leads to both acantholysis and apoptosis resulting in acantholytic dyskeratosis affecting the cell adhesion complexes. The links for the mechanism(s) behind the periodontal complications have not been documented.
5. Fibrous cemento-osseous dysplasia	20.8	A disease which produces jaw lesions close to where the teeth are formed. Areas of normal bone are replaced by benign connective tissue matrix that displays varying degrees of mineralisation in the form of woven bone or cementum-like acellular basophilic structures. The mechanism(s) behind the periodontal complications have not been documented, however, as the cells of origin are derived from the periodontal ligament this may be relevant to the disease process.
6. Acute porphyria	18.8	A metabolic disorder that is characterised by partial deficiency of the enzyme hydroxymethylbilane synthase. The enzyme deficiency results in the accumulation of porphyrin precursors in the body. This is caused by a mutation in the *HMBS* gene which is inherited as an autosomal dominant trait. Together with other eliciting factors it can cause abdominal pain, constipation, atachycardia, hypertension, behavioral changes, seizures, and peripheral neuropathy with marked muscle weakness and paralysis. The periodontal connection has not been established. Possibly it is due to the build up porhyrin in the tissues or muscle weakness and paralysis.
7. Spondyloepiphyseal/-metaphyseal dysplasia	18.2	Spondyloepiphyseal dysplasia congenita (SEDC) is a rare genetic disorder characterised by prenatal deformities such as skeletal and joint malformations of the spine, hips, and knees and abnormalities affecting the eyes. Such growth deformities lead to short stature. SEDC is caused by mutations in the type II collagen (*COL2A1*) gene. The disorder is inherited in an autosomal dominant manner, but most cases are de novo mutation with no previous family history. The periodontal connection is not known.
8. Inclusion body myositis (IBM)	17.9	IBM is characterised by chronic, progressive muscle inflammation accompanied by muscle weakness. There may be weakness of the wrist and finger muscles and atrophy of muscle bulk in the arms and legs. Difficulty swallowing occurs in approximately half of IBM cases. Symptoms of the disease usually begin after the age of 50. The connection with periodontal complications is not known but it may be due to muscle weakness interfering with oral hygiene measures.
9. Facioscapulohumeral muscular dystrophy	17.6	A genetically acquired disease (autosomal dominant) which leads to progressive muscle weakness and severely decreased functional capacity. The muscles involved include the facial, shoulder girdle, and lower extremities. It usually starts in the face and spreads to the other muscles. A number of patients also have hearing loss and retinovasculopathy. The cause of the disease is an inappropriate expression of the DUX4 protein due to the interaction with the chromosal D4Z4 macrosatellite. No previous connections with periodontal complications have been reported. One possible connection could be the inability to perform adequate oral hygiene due to the disability.
10. Arthrogryposis multiplex congenita (AMC)	17.4	AMC is a development of multiple joint contractures affecting 2 or more areas of the body prior to birth. This means that a joint becomes permanently fixed in a bent or straightened position, which can impact the function and range of motion of the joint and may lead to muscle atrophy. The periodontal connection is not known but could be due to a physical disability.
11. Rare chromosome or genetic defects	17.2	These are a collection of rare syndromes, defects, and diseases, for example, trisomy 16, Edwards syndrome, cat eye syndrome, cri du chat syndrome, Patau syndrome, triple X syndrome, Williams syndrome, 22q11.2 deletion syndrome, Turner syndrome, Wolf-Hirschhorn syndrome, Jacobsen syndrome, and Angelman syndrome. No common mechanism for the periodontal complications of these defects have been identified. However, case reports have identified periodontal involvements for some of these, for example, cri du chat syndrome.
12. Friedreich's ataxia	16.7	An autosomal recessive disease that causes neurodegeneration. It results in muscle weakness and loss of sensation and proprioception, causing problems with movement as well as impaired speech. The symptoms tend to worsen as time progresses, so many patients end up in wheelchairs, lose their vision and hearing, and experience other medical complications such as diabetes mellitus and scoliosis. The cause of the disease is a reduction in frataxin, which is necessary for the production of mitochondrial adenosine triphosphate (ATP) and the management of iron stores. No previous connections with periodontal complications have been reported; however, diabetes is a known risk factor. In addition, the inability to perform adequate oral hygiene due to the disability may be an important risk factor.
13. Bullous mucous membrane pemphigoid	16.7	This is due to autoantibodies targeting components of the basement membrane zone (hemidesmosomal proteins BP180 and BP230). It is characterised by blisters, erosions, erythema, and pruritic urticarial plaques on the whole body. It does exhibit the Nikolsky sign. This means that rubbing of the skin does not lead to intraepidermal separation. Oral manifistations consist of localised or generalised recurrent blisters in addition to gingival inflammation and severe alveolar bone loss that may be localised or generalised.
14. Churg-Strauss syndrome	16.7	Churg-Strauss syndrome (eosinophilic granulomatosis with polyangiitis) is a specific variant of the group of diseases characterised by necrotising vasculitis of small and medium-sized systemic blood vessels. Other subtypes within the broad group include Wegener's granulomatosis/granulomatosis with polyangiitis, microscopic polyangiitis, and polyarteritis nodosa. It is distinctive from the other diseases in the category of the coexistence of asthma, rhinosinusitis, and the presence of peripheral eosinophilia. The connection with periodontal destruction is likely to be due to a defective phagocytosis and intracellular killing of microorganisms.
15. Pierre Robin sequence	16.7	Pierre Robin sequence consists of micrognathia, glossoptosis, and airway obstruction. Infants frequently present at birth with a hypoplastic mandible and difficulty breathing. The smaller mandible displaces the tongue posteriorly, resulting in obstruction of the airway. No direct mechanism for periodontal complications have been described, but the anatomical abnormalities may make it difficult for adequate oral hygiene.
16. Multiple endocrine neoplasia type 1 (MEN-1)	15.8	An autosomal-dominant disorder characterised by tumours of the parathyroids, pancreas, and anterior pituitary. The cause of the disease is a mutation in the *MEN1* gene which interferes with cell division, proliferation, transcriptional regulation, and genome stability. No direct mechanisms explaining the periodontal complications exist. However, interference with the hormone production from 3 such important glands, it should provide a number of possible links to explain these mechanisms. For example, in hyperparathyroidism widening of the periodontal ligament and osteolytic lesions in the jaws have been described.
17. Cerebellar ataxia	15.4	Cerebellar ataxia is an inability to coordinate muscle movement especially of the hands and legs due to disease or injury to the cerebellum. This is the area in the brain that controls muscle movement. The periodontal connection is likely due to the problem with muscle coordination making oral hygiene difficult.
Wegener's granulomatosis	12.1	A necrotising granulomatous inflammation coupled with small and medium-sized blood vessel vasculitis caused by antineutrophil cytoplasmic antibodies antibodies involving the respiratory tract, kidneys, and nasal and sinus regions. The connection with periodontal destruction is likely to be due to a defective phagocytosis and intracellular killing of microorganisms. The diagnosis is done by genetic testing for mutations in genes encoding for gp91phox, p47phox, p22phox, p67phox, and p40phox.
Hypophosphatemic rickets	11.5	It is caused by mutations (dominant and recessive) in fibroblast growth factor 23 (*FGF23*). This gene influences mineral ion homeostasis and leads to alteration of bone and tooth mineralisation and cementum structure. In terms of periodontal complications, a mutation in the X-chromosomal PHEX gene has been suggested as being the most likely. The periodontal complications are thought to be due to faulty bone regeneration and cementum hypoplasia. The following must be present for a diagnosis: increased serum alkaline decreased serum phosphate levels phosphatase, normal serum calcium, and normal serum parathyroid hormone.
Ehlers-Danlos syndrome (EDS)	10.0	EDS disorders are caused by mutations in genes encoding fibrillary collagens or enzymes involved in the biosynthesis of these. The patients often have hyperpigmentation, bruise easily, and have joint hypermobility. Periodontal destruction with loss of teeth is most often fount in type VIII EDS, but also to a lesser extent in types I and IV. Gingival recession is a common feature. The periodontal destructions can start as early as in the teens. The diagnosis of the periodontal EDS is made by a molecular genetic analysis identifying specific heterozygous mutations in the *C1S* or *C1R* genes.

The relatively low proportion of patients treated for periodontal diseases in each of the RD categories was an interesting observation. The impression given in case reports and reviews is that periodontal complications occur in most or all of these patients due to the periodontal-systemic connection. This is clearly not the case in the present findings.

As this was a novel study with no external studies for verification, it was important to assess the validity of the data. It was particularly important to confirm the treatment expectations for certain “well-known” RDs and also to establish whether the treatments represented the real-world situation for the patients. The first point was assessed by a number of RDs which had been presented in major review articles including glycogenesis, Papillon-Lefèvre syndrome, neutropaenia, Wegener's granulomatosis, hypophosphatemic rickets, Ehlers-Danlos syndrome, and epidermolysis bullosa dystrophic.^7^^,^^8^ These all received higher levels of treatment in the present study. In addition, the study also combined the levels of periodontal treatment with the levels of treatment performed by periodontal specialists to identify the RDs with more severe periodontal complications. The fact that neutropaenia rated highest in this treatment prevalence supported the long-held knowledge that neutrophil disorders are associated with severe periodontal diseases.

Regarding the real-world treatment of these patients, it was important to discern whether the patient selection could be biased because of the higher economic benefits they received, that is, an overtreatment, resulting in too many patients receiving treatment. This is not likely, as only 135 RDs out of 357 RDs and 10.2% of the total patients with such economic benefits received treatments. Finally, conversely, it was important to establish whether patients with a treatment need actually received treatment. A lack of required treatment would likely have been due to a lack of knowledge of their rights in the National Health System, a dental supply shortage, or a generally low dental attendance in the population. These do not seem to be likely for the following reasons: When patients are diagnosed with RDs in Norway, they undergo counselling, where they receive information about the disease and their rights under the Norwegian National Insurance Scheme. They are instructed to inform their dentist about their diagnosis and their rights for monetary support for dental treatment.

Access to dental treatment is good.^14^^,^^15^ The number of dentists in relation to the population is higher in Norway than in most countries in the world. Dentists are distributed evenly according to the regions. Finally, more than 80% of patients attend their dentist every year, with an additional 10% attending every other year.^16^ These data, however, apply to the general population and not necessarily to the patients with RDs in the present observation.

Overall, the data and the results thus seem to be a valid representation of the treatments performed for patients with RDs in Norway.

Limitations of this observational study include the following: Information is only available on patients older than 20 years and those who are not institutionalised. The present study thus applies to patients with RDs who are functioning sufficiently well in society to be treated in private dental practice settings. There is a possibility that national variations in the RDs exist, including clustering of certain disorders in areas or countries. This may complicate generalisations of the present results, especially as no other equivalent studies can be used for comparison or verification. Further, the present study is cross-sectional and covers 1 year only. It is possible that a more of a longitudinal study could have elicited more detailed information. However, the 2 previous years were also assessed and showed nearly identical treatment levels.

In addition, no information is included on confounders, such as the levels of oral hygiene, smoking, or other systemic diseases. As this is a preliminary observational study, no statistics has been applied to the results apart from levels of treatment in percentages. A follow-up study would require a robust statistical setup, including factors like intra-/interexaminer reliability, intraclass coefficient, a test unit, stratification of the results on the basis of a stepwise analysis adding initial PD and PISA of patients, confounders, and a power sample analysis.

## Conclusion

A large number of people living with RDs have the extra burden of also being susceptible to periodontal diseases. The analysis in the present study was carried out on a population register data from Norway, and the results showed that 10.2% of all patients with RDs and patients with 135 RDs received periodontal treatments. The RDs represented a large variety of conditions, syndromes, and diseases. Within each group, there was a range in the level of treatments performed. The mechanisms that affected the periodontal tissues were complicated and varied and did not affect all patients within an RD group (range, 1%–28.6%). Many of the diseases which received treatments had no previously described connections to periodontal diseases, and thus a number of periodontal-systemic mechanisms have yet to be uncovered. Physical and mental disabilities as part of some RDs may affect oral health measures and contribute to increased periodontal treatment needs.

## Author contributions

Ø. Fardal, I. Skau, and J. Grytten all contributed to conception, design, and data acquisition and interpretation; performed all statistical analyses; and drafted and critically revised the manuscript. In addition, I. Skau and J. Grytten contributed to data acquisition.

## Conflict of interest

None disclosed.
